# Model-based integration of chimeric antigen receptor T-cell kinetics and tumor features to predict therapeutic outcomes in patients with B-cell malignancies

**DOI:** 10.3389/fimmu.2026.1927857

**Published:** 2026-10-02

**Authors:** Jonas Schadt, Maximilian Alexander Röhnert, Paul Matthiensen, Uta Oelschlägel, Martin Bornhäuser, Frank Kroschinsky, Raphael Teipel, Ingmar Glauche, Malte von Bonin, Artur César Fassoni

**Affiliations:** 1 Department of Medicine I, Faculty of Medicine and University Hospital Carl Gustav Carus, TUD Dresden University of Technology, Dresden, Germany; 2 Institute for Medical Informatics and Biometry (IMB), Faculty of Medicine Carl Gustav Carus, TUD Dresden University of Technology, Dresden, Germany; 3 Instituto de Matemática e Computação, Universidade Federal de Itajubá, Itajubá, Minas Gerais, Brazil

**Keywords:** antigen density, B-cell lymphoma, CAR T-cell kinetics, mathematical model, ordinary differential equations, predictive biomarkers, treatment outcome, tumor burden

## Abstract

**Introduction:**

Chimeric antigen receptor (CAR) T-cell therapy is a standard treatment for several hematological malignancies, yet patient-specific responses and toxicities remain hard to predict. Although multiple covariates and risk scores exist, their prognostic accuracy remains limited.

**Methods:**

We prospectively measured CAR T-cell kinetics in peripheral blood of 92 patients with B-cell malignancies using multicolor flow cytometry. To explore underlying mechanisms, we developed a mechanistic model of CAR T-cell and tumor cell interactions.

**Results:**

We captured four phases of CAR T-cell kinetics in peripheral blood: distribution, expansion, contraction, and persistence. Patients with weak expansion or deep contraction are more likely to relapse. When fitted to the 46 patients with suDicient data points, the model reproduces individual CAR T-cell kinetics but fails to predict outcomes using uniform tumor parameters. We demonstrate that identical CAR T-cell dynamics may result from diDerent tumor features, including growth rate, initial burden, or antigen density. Accurately estimating these tumor-specific factors may improve relapse prediction, while common surrogates like blood lactate dehydrogenase, International Prognostic Index score, or Ann-Arbor stage do not.

**Conclusion:**

We conclude that CAR T-cell kinetics within the first month can serve as early prognostic markers. Integrating tumor burden estimates is required to reconcile kinetics with individual outcomes and may, upon prospective validation, further improve risk stratification in patients with B-cell malignancies receiving CAR T-cell therapy.

## 1 Introduction

Chimeric Antigen Receptor T-cell (CAR T-cell) therapy represents a significant advancement in the field of immunotherapy and offers a curative perspective for hard-to-treat malignancies. It involves the genetic modification of autologous T-cells to express a chimeric antigen receptor (CAR), enabling recognition and binding of tumor-associated proteins on malignant cells, thereby mediating their effective elimination. This targeted approach has shown remarkable success in treating hematological malignancies such as B-cell acute lymphoblastic leukemia (B-ALL), non-Hodgkin lymphomas (B-NHL), and multiple myeloma (MM) (1).

Despite its success, CAR T-cell therapy faces several clinical challenges. While some patients do not respond to treatment, others experience disease relapse after initial response. Additionally, severe side effects, including cytokine release syndrome (CRS), immune effector cell-associated neurotoxicity syndrome (ICANS), and immune effector cell associated hematotoxicity (ICAHT), contribute to non-relapse mortality (2).

The systematic assessment of factors influencing CAR T-cell therapy remains challenging due to substantial variability across diseases, patient histories, and treatment protocols (3, 4). Disease-specific differences, such as tumor microenvironment and antigen expression, may affect CAR T-cell function. Heterogeneous treatment histories may further influence immune status, chemosensitivity, or tumor burden. Variability also arises from differences in CAR T-cell design (e.g. costimulatory domain), manufacturing processes, patient selection and toxicity management. Together, these factors lead to heterogeneous clinical data and complicate consistent evaluations of potential predictive factors for therapy efficacy and safety.

Based on an increasing number of clinical datasets, mechanistic mathematical models have emerged as powerful tools to identify common treatment patterns and to understand complex interactions shaping the response to CAR T-cell therapy (5–20). Time-resolved measurements of CAR T-cell kinetics and tumor burden are essential for constructing and validating models that functionally link T-cell expansion, tumor targeting and T-cell exhaustion. The resulting models can explain typical response patterns and support predictions of clinical outcomes. We have recently shown that the integration of cytokine release dynamics following CAR T-cell infusion can help to identify strategies for the prevention of CRS (21).

Here, we aim to determine whether CAR T-cell kinetics measured by multicolor flow cytometry in peripheral blood at sparse time points, combined with standard clinical indicators of tumor burden, can elucidate the functional interactions driving therapy response and predict treatment outcome and toxicity. By decomposing CAR T-cell dynamics into four distinct phases (distribution, expansion, contraction, and persistence) we hypothesize that specific kinetic features, such as expansion rate, peak cell levels, and contraction depth, are predictive of therapeutic success or failure. We further analyze the association between these kinetic features and the occurrence of side effects.

To uncover the mechanistic basis of response heterogeneity, we employ an ordinary differential equation (ODE) model that explicitly links CAR T-cell kinetics to tumor characteristics, including growth rate and initial tumor load. Within this framework, we address the inverse problem: inferring hidden tumor-specific parameters from early CAR T-cell dynamics to explain why patients with similar kinetic profiles can experience divergent clinical outcomes. This patient-specific mathematical strategy allows us to identify the tumor-intrinsic factors that most strongly condition long-term efficacy. Additionally, we investigate whether the integration of CAR T-cell kinetics with clinical surrogate markers, such as lactate dehydrogenase (LDH) levels, International Prognostic Index (IPI) score, and Ann-Arbor stage, improves outcome prediction.

Taken together, this study provides a mechanistic proof-of-concept that early, clinically accessible kinetic data can inform a mathematical model capable of inferring unobserved tumor characteristics essential for predicting the long-term efficacy of CAR T-cell therapy.

## 2 Materials and methods

### 2.1 Patient data

We collected data from 92 consecutive patients treated in-label with an anti-CD19 directed CAR T-cell therapy (axicabtagen ciloleucel (axi-cel), brexucabtagene autoleucel (brexu-cel), tisagenlecleucel (tisa-cel), lisocabtagen maraleucel (liso-cel)) for B-cell neoplasms at the University Hospital Carl Gustav Carus (Dresden, Germany) between February 2021 and June 2025. The dataset included clinical parameters (e.g. disease status, side effects, tumor response, see **Supplementary Data Table 1**). Patients were censored after having progressive disease or receiving any new antineoplastic therapy. Median follow up was 472 days (interquartile range 206 to 611 days).

The study was approved by the institutional review board at TUD Dresden University of Technology (IRB00001473, reference number BO-EK-66022024) and informed written consent was obtained from all participants. All procedures involving patients or patient data were performed in accordance with the Declaration of Helsinki.

### 2.2 CAR T-cell kinetics by multicolor flow cytometry

CAR T-cell kinetics were measured at multiple time points in the PB by MFC on a BD FACSCanto™ II (Becton Dickinson, Franklin Lakes, NJ, USA). CAR T-cells were detected by fluorochrome coupled antibodies targeting CD45 (BD Biosciences, Cat# 560777, Clone HI30, 5µl) and CD3 (BD Biosciences, Cat# 345764, Clone SK7, 5µl), as well as a biotinylated CD19-based CAR detection reagent (Miltenyi Biotec, Cat# 130-129-550, 2µl) together with a fluorochrome coupled Biotin antibody (Miltenyi Biotec, Cat# 130-111-068, Clone REA746, 2µl). To enhance accuracy, a control measurement without CAR detection reagent was performed for each sample according to the Fluorescence Minus One (FMO) concept (see **Supplementary Figure S1** for details on the gating strategy). This allowed us to determine the percentage of CAR T-cells among leukocytes. Absolute CAR T-cell counts were calculated from these values using the differential blood count (Sysmex, Kobe, Japan, XN-1000). If the MFC measurement detected no CAR T-cells or a number below the detection threshold of reliable quantification, we assigned an undetectable value based on the individual detection limit for that time point (primarily depended on the total leukocyte count). The initial CAR T-cell count at infusion was set to the dose specified by the manufacturer.

### 2.3 Multiphasic segmentation of CAR T-cell kinetics

We developed an automated procedure to segment the kinetics of individual CAR T-cell time courses in PB into four phases (22, 23): distribution, expansion, contraction, and persistence (**Figures 1D, E**). Using the time courses of each patient as inputs, the routine generates a set of eight patient-specific segmentation parameters characterizing the four phases with a slope (s_D_, s_E_, s_C_, s_P_) and corresponding CAR T-cell counts at specific time points within each phase (C_min_, C_max_, C_con_, C_per_). The input is the individual time course containing n absolute CAR T-cell counts C(t_i_) at days t_i_ plus the CAR T-cell dose at the day of infusion (t_0_, C(t_0_)). In the first step, the time of CAR T-cell peak is set to t_max_=argmax{C(t_i_), i=1,…, n} (the initial dose is excluded), and the CAR T-cell peak is defined as C_max_=C(t_max_). In the next step, the CAR T-cell minimum before the peak is identified at t_min_=argmin{C(t_i_), t_0_≤t_i_≤t_max_} with cell count C_min_=C(t_min_). If t_min_=t_max_, then C_0_>C_max_ without an expansion phase; two patients fell into this category and were excluded from the subsequent analysis (**Figure 1A**). If t_min_<t_max_, the expansion phase is defined by the straight line connecting (t_min_, log C_min_) to (t_max_, log C_max_), with slope denoted by s_E_. Then, if t_min_=t_0_, the patient did not have an observable distribution phase. If t_min_>t_0_, the distribution phase is defined by the straight line connecting (t_0_, log C(t_0_)) to (t_min_, log C_min_), with slope denoted by s_D_. The contraction and persistence phases are then defined as follows. If t_max_=t_n_, these two phases are not observed for the patient. If t_max_<t_n_, at least a contraction phase is observed. We define an auxiliary straight-line h(t) connecting the peak point (t_max_, log C_max_) and the final point (t_n_, log C(t_n_)), given by

**Figure 1:**
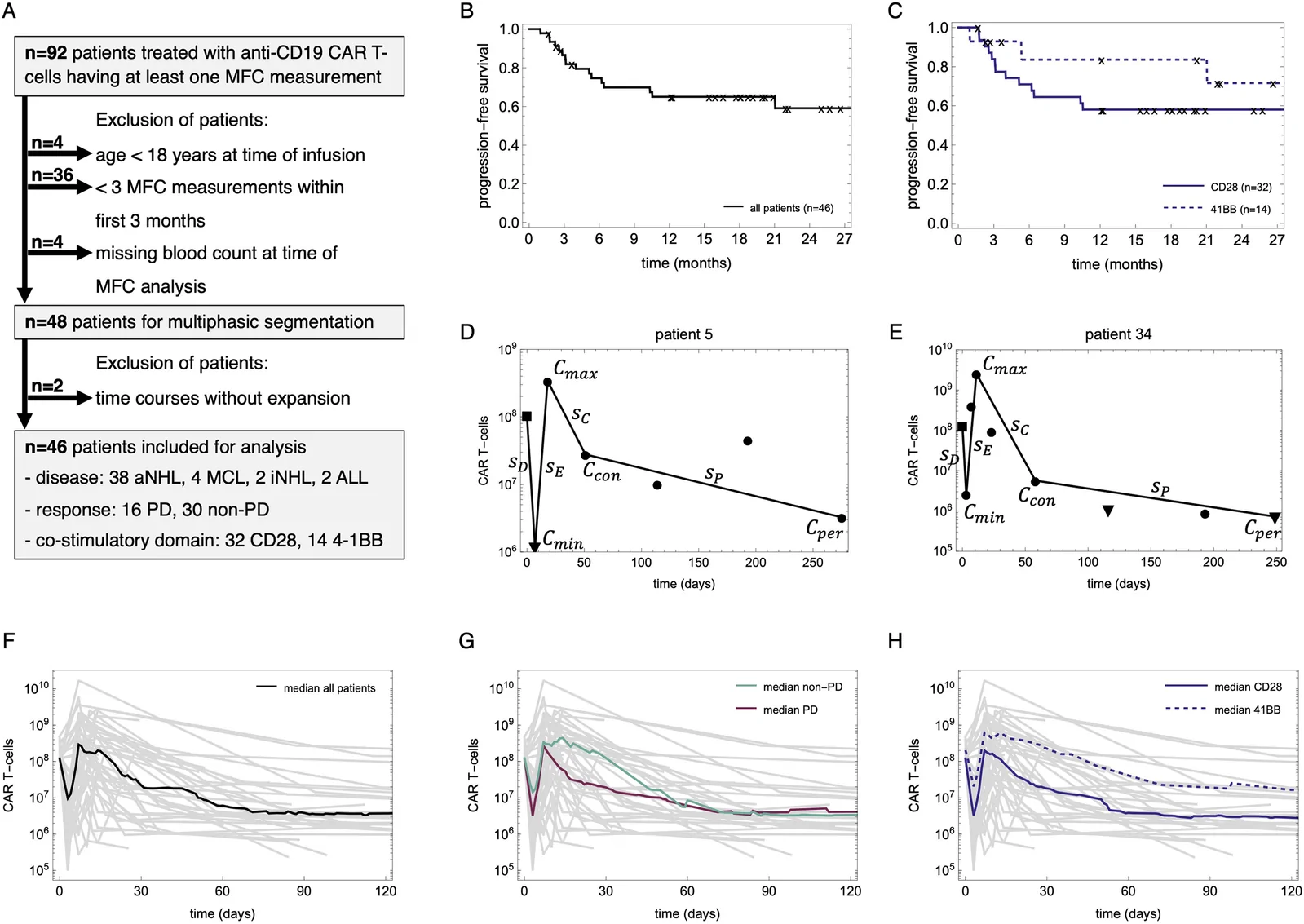
Study design, patient cohorts, and CAR T-cell kinetics. **(A)** A total of 92 patients treated with anti-CD19 Chimeric antigen receptor (CAR) T-cells at the University Hospital Carl Gustav Carus, Dresden were screened for data availability, with 46 patients meeting the inclusion criteria for analysis. **(B)** Progression-free survival curve illustrating therapeutic outcome: 16 patients exhibited disease progression (PD), while 30 showed no progression (non-PD), including complete remission (CR), partial remission (PR) or stable disease (SD) during available follow up. **(C)** Progression-free survival stratified by CAR T-cell construct: 32 patients received CD28-based CAR T-cells, and 14 received 4-1BB-based CAR T-cells. **(D, E)** Representative examples of individual CAR T-cell time courses and segmentation into four kinetic phases characterized by distinct slopes (s_D_ for distribution, s_E_ for expansion, s_C_ for contraction, and s_P_ for persistence) and key levels, namely, CAR T-cell minimum after infusion (C_min_), peak after expansion (C_max_), levels at the end of contraction (C_con_) and persistence phases (C_per_). Circles represent measured cell numbers while triangles are set at the individual detection threshold representing measurements with cell numbers below. **(F-H)** Median CAR T-cell kinetic profiles for distinct patient cohorts: all patients **(F)**, non-PD vs. PD patients **(G)**, and CD28 vs. 4-1BB costimulatory domains **(H)**.

(1) $$h\left(t\right)=\log C_{\max}+\frac{\left(\log C_{\text{n}}-\log C_{\max}\right)}{\left(t_{\text{n}}-t_{\max}\right)}\left(t-t_{\max}\right).$$

The point defining the transition between the contraction and the persistence phase is defined by the point t_i_ where the difference between h(t_i_) (Equation 1) and log C(t_i_) is maximum, i.e., t_con_=argmax{h(t_i_) – log c(t_i_), t_max_<t_i_≤t_n_}, with corresponding CAR T-cell count C_con_=C(t_con_). The contraction phase is then defined by the straight line connecting (t_max_, log C_max_) to (t_con_, log C_con_), with slope denoted by s_C_. If t_con_=t_n_, the persistence phase is not observable. If t_con_<t_n_, the persistence phase is defined by the straight line connecting (t_con_, log C_con_) to (t_n_, log C_n_) with slope s_P_; the persistence level is C_per_=C(t_n_).

### 2.4 Statistics and reproducibility

Analyses were performed in *Wolfram Mathematica* (Wolfram Research, Inc., Champaign, IL, USA). Cox proportional hazards models were fitted using *CoxModelFit* to assess progression-free-survival (PFS) across subgroups. Group comparisons of continuous covariates were conducted using the Mann–Whitney U test (*MannWhitneyTest*). Statistical significance was defined as α = 0.05. Of the 92 patients initially screened, 46 were not eligible for this analysis on the basis of clinical eligibility (age< 18 years at infusion, n=4), data availability (fewer than three MFC measurements within the first three months, n=36; missing blood count at the time of MFC analysis, n=4), or a constraint intrinsic to the segmentation procedure (time courses without an identifiable expansion phase, n=2) (**Figure 1A**); no patient was excluded based on clinical outcome. The final cohort (n=46) represents the complete available dataset suitable for kinetic analysis and is used throughout **Figures 1B, C, F–H**, **2A–F**, **3B, C**, **4G–I**, **5A, B, F**.

**Figure 2:**
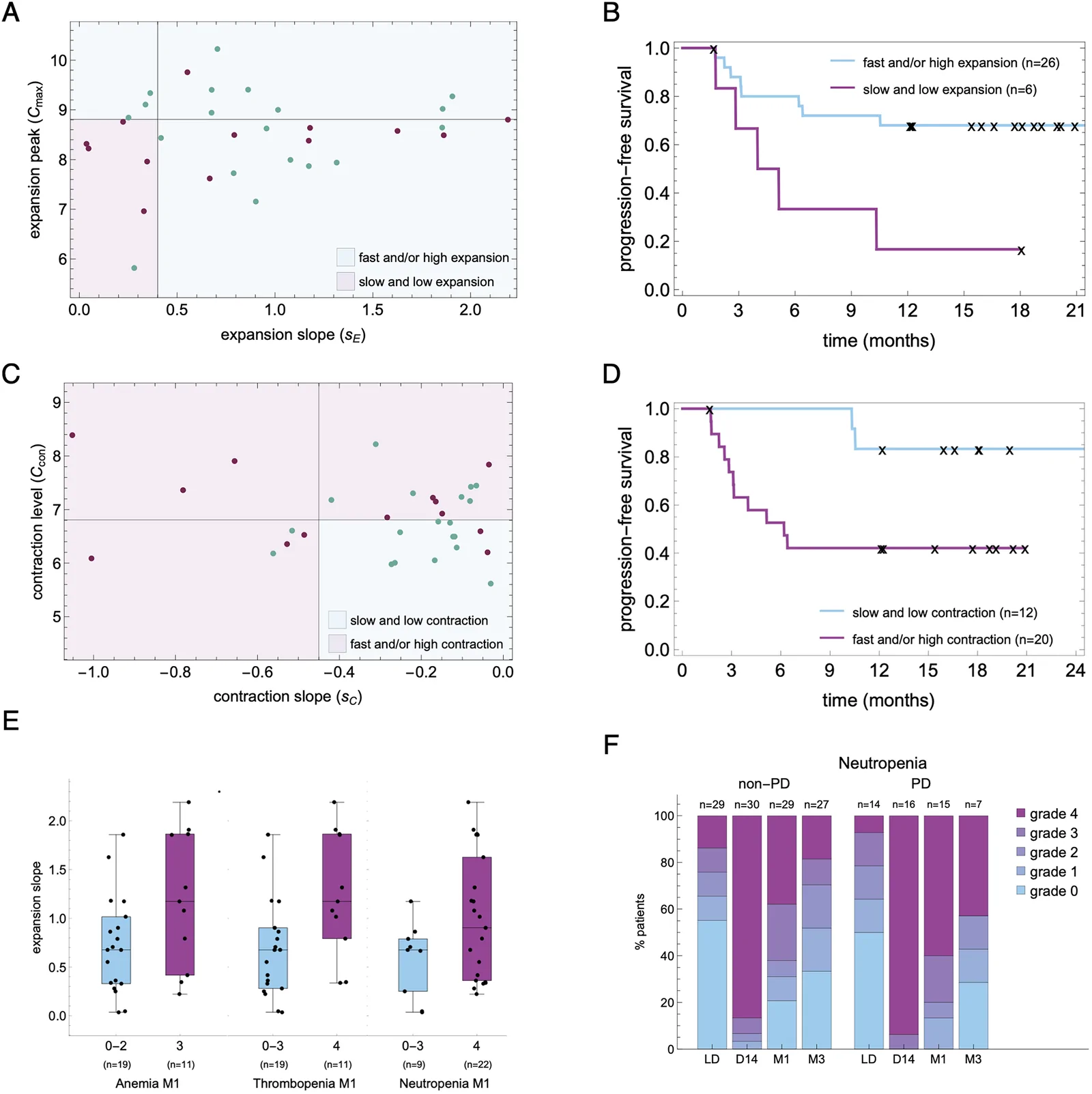
Associations between CAR T-cell kinetics, effectivity, and cytopenias. **(A–D)** Analysis of Chimeric antigen receptor (CAR) CAR T-cell kinetic parameters during expansion and contraction phases. **(A, B)** Higher peak CAR T-cell concentration (C_max_ > 8.8) and/or steeper slopes during the expansion phase (s_E_ > 0.4) are associated with better therapeutic outcomes. Patients with both low C_max_ and slow s_E_ predominantly ended with progressive disease (PD). **(C, D)** Slower contraction slopes (s_C_ > -0.45) and lower contraction values (C_con_< 6.8) are associated with non-PD. **(E)** Association between CAR T-cell expansion slope and cytopenia at month 1. Data were grouped according to CTCAE v5.0 and clinical relevance (transfusion or application of growth factors). **(F)** Evolution of cytopenia in non-PD and PD group, showing the maximum grades of neutropenia over time (anemia, thrombopenia in **Supplementary Figure S4B**). While there are no differences in baseline cytopenia between PD and non-PD groups, the PD group shows persistent cytopenia with higher severity up to month 1 and month 3.

**Figure 3:**
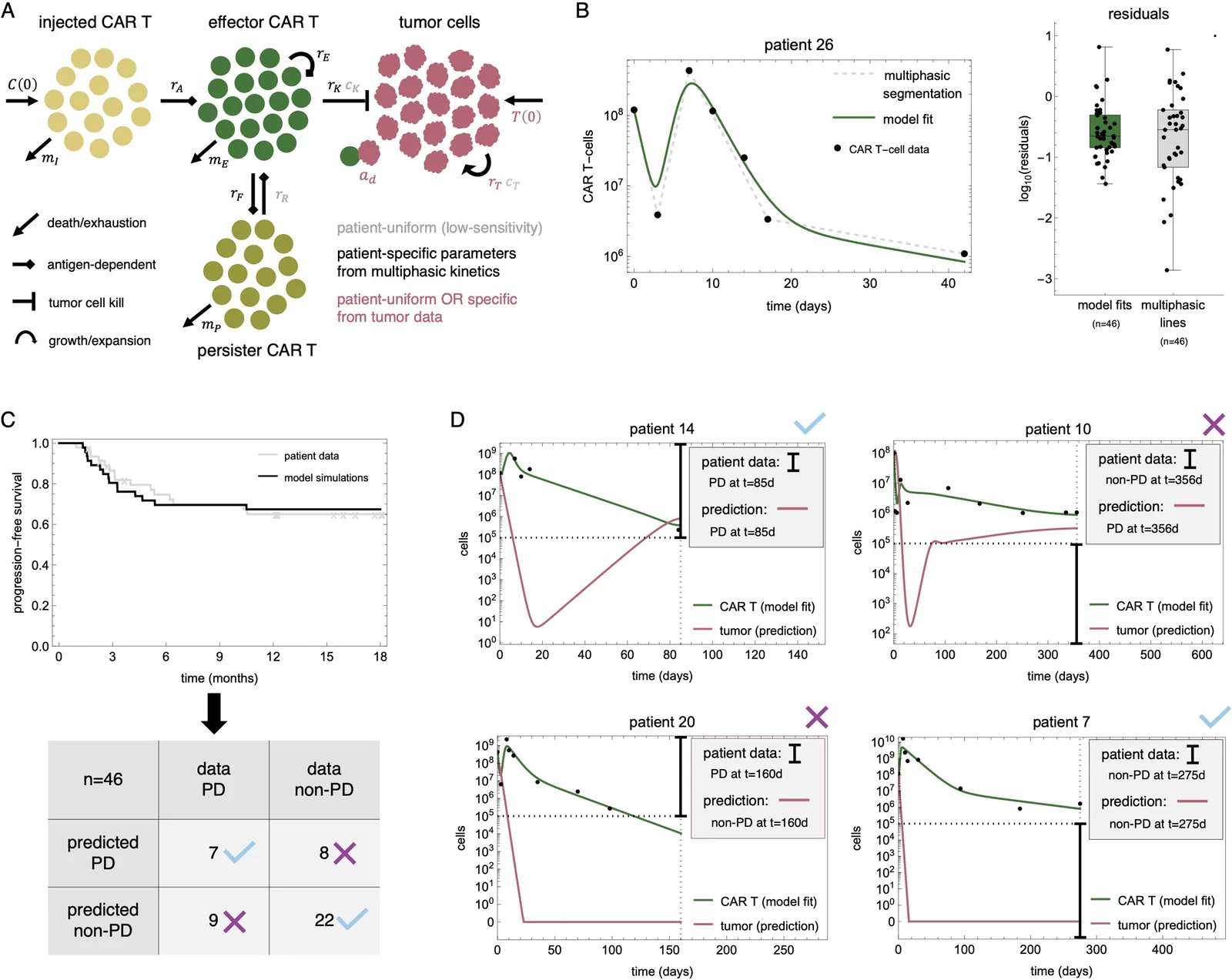
Mechanistic modeling of CAR T-cell kinetics and tumor response across patients. **(A)** Schematic of the compartmental model describing Chimeric antigen receptor (CAR) T-cell kinetics and tumor dynamics. **(B)** Left: model fit (green) compared to multiphasic segmentation (dashed gray) for a representative patient, capturing the multiphasic CAR T-cell behavior. Right: residual comparison shows that model fits perform as well as multiphasic segmentation, demonstrating alignment between the model and observed CAR T-cell data at the individual level. **(C)** Top: model-predicted progression-free survival correlates well with clinical outcomes at the population level. Down: using uniform tumor parameters results in inaccurate predictions of individual efficacy, including false-negative and false-positive outcomes (off-diagonals in 2x2 table). **(D)** Panels illustrate these diverse patient response patterns, with correct and wrong predictions. The green line represents the ODE model fit of the CAR T-cell kinetics. The red line shows the hypothesized tumor dynamics. The clinical response of each individual patient at the last follow-up time is indicated by the vertical bars (above the dashed line for progressive disease (PD), below the dashed line non-PD) and compared with model-predicted tumor responses (hypothesized tumor dynamics end above the dashed line for PD, hypothesized tumor dynamics end below the dashed line for non-PD).

**Figure 4:**
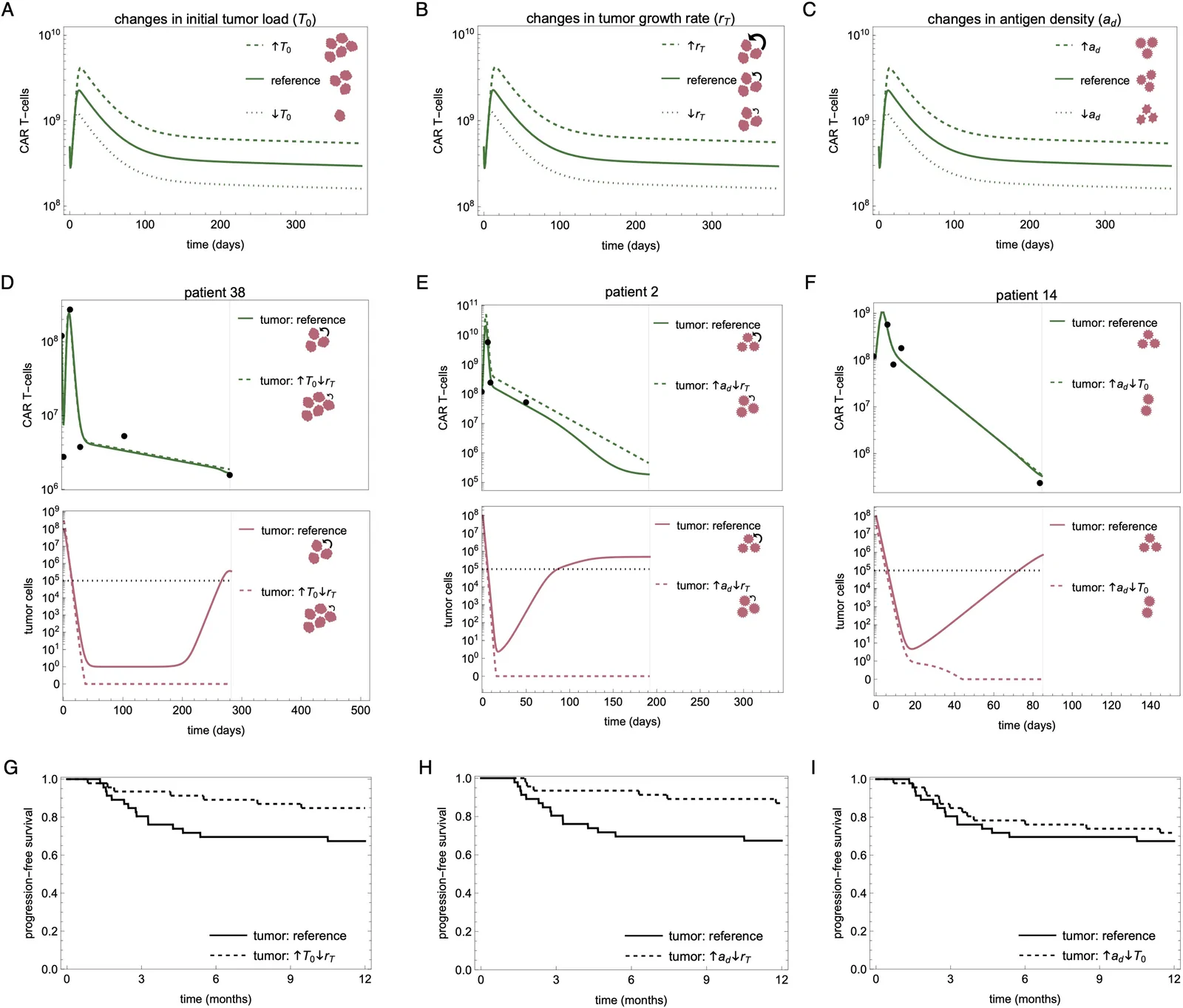
Tumor heterogeneity explains identical CAR T-cell kinetics with variable tumor outcome. **(A–C)** Increasing one tumor parameter (T_0_, r_T_, or a_d_) in silico leads to higher Chimeric antigen receptor (CAR) T-cell peaks, while decreasing the parameters results in lower peaks. **(D–F)** Fitting the model to individual CAR T-cell data can be achieved by varying two tumor parameters in opposite directions, leading to nearly indistinguishable CAR T-cell kinetics but different tumor outcomes. Although they result from the same CAR T-cell kinetics, the simulated tumor outcomes range from ongoing remission (non-PD) to relapse (PD). **(G, H)** Higher tumor load or antigen density combined with lower tumor growth rate improve therapeutic outcomes in silico compared to reference values. **(I)** Combining changes in antigen density and tumor load preserves the overall outcome in silico.

**Figure 5:**
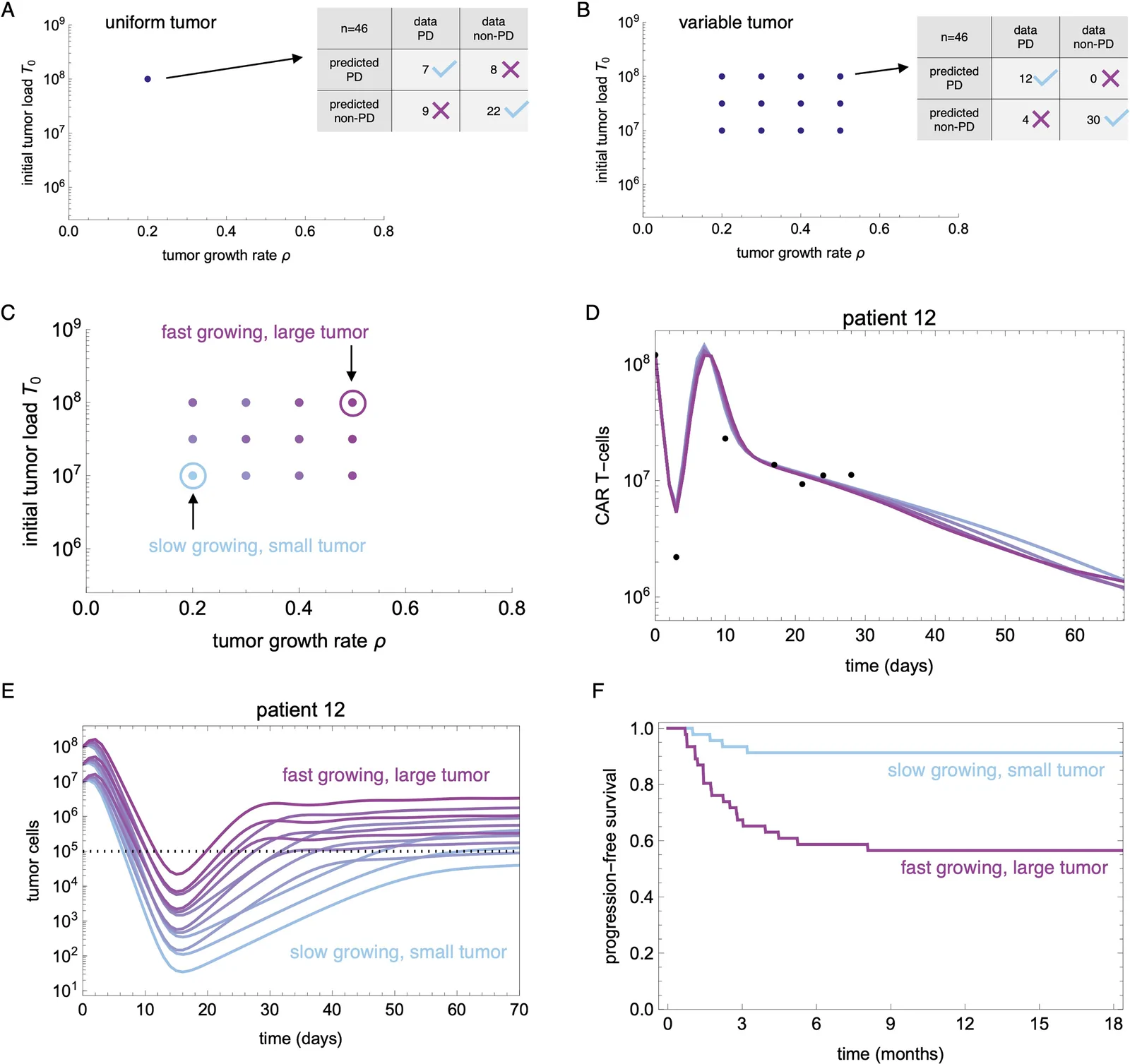
Prediction of therapy response with variable tumor growth rate and initial tumor load. **(A)** Assuming uniform population-level values for tumor growth rate and initial tumor load prevents accurate therapy response prediction. **(B)** Incorporating heterogeneity in these parameters significantly improves the model’s predictive accuracy, reducing misclassifications to only four false negatives. **(C)** To further assess the impact of these parameters, we tested each combination individually across the entire cohort, simulating a range of tumor characteristics. **(D, E)** While all parameter combinations result in similar Chimeric antigen receptor (CAR) T-cell expansion dynamics **(D)**, they lead to distinct tumor response predictions, with smaller, slower-growing tumors correlating with better clinical outcomes **(E)**. **(F)** At the population level, each parameter pair in the grid leads to different predicted progression-free survival (PFS) rates at 18 months. Simulating the smallest and slowest-growing tumor results in a predicted PFS of 0.9, whereas the largest and fastest-growing tumor corresponds to a predicted PFS of 0.5.

### 2.5 ODE model

Building on our previous work (21) and classical models of T-cell response (24–27), we developed a mechanistic model based on ODEs to describe CAR T-cell therapy and tumor response. The model describes the interaction between CAR T-cells and antigen-expressing tumor cells (T). Through antigen contact, injected CAR T-cells (C_I_) are activated (expander CAR T-cells, C_E_), leading to an increasing total CAR T-cell concentration. These activated CAR T-cells can target tumor cells, while in the absence of antigen contact, they transform into persistent, non-targeting CAR T-cells (C_P_) (**Figure 3A**). These three mentioned CAR T-cell populations represent conceptual populations rather than clearly biologically distinguishable cell populations. Accordingly, they cannot be identified individually by MFC. Only the total number of CAR T-cells were measured in the PB. The system is governed by:

(2) $$\frac{dC_{I}}{dt}=-r_{A}F\left(T\right)C_{I}-m_{I}C_{I}$$

(3) $$\frac{dC_{E}}{dt}=r_{A}F\left(T\right)C_{I}+r_{E}F\left(T\right)C_{E}-r_{F}\left(1-F\left(T\right)\right)C_{E}+r_{R}F\left(T\right)C_{P}-m_{E}C_{E}$$

(4) $$\frac{dC_{P}}{dt}\;=r_{F}\left(1-F\left(T\right)\right)C_{E}-r_{R}F\left(T\right)C_{P}-m_{P}C_{P}$$

(5) $$\frac{dT}{dt}=r_{T}T\left(1-\frac{T}{c_{T}}\right)\left(\frac{T-1}{T+1}\right)-r_{K}\frac{C_{E}}{c_{K}+C_{E}}T$$

The model incorporates compartment-specific death rates (m_I_, m_E_, m_P_), with m_E_ also encompassing exhaustion. Antigen-dependent transitions are modulated by the Michaelis-Menten function $\text{F}\left(\text{T}\right)=\text{T}/\left(\text{T}+{{\text{a}}_{\text{d}}}^{-1}\right)$. Here, a_d_ represents antigen density of tumor cells, such that a higher a_d_ lowers the half-saturation constant ${{\text{a}}_{\text{d}}}^{-1}$, increasing activation sensitivity. F(T) modulates activation of injected CAR T-cells with rate r_A_, expansion of activated CAR T-cells with rate r_E_, recruitment of persister cells with rate r_R_, while inhibiting the transition to persistence with rate r_F_. Tumor cells follow logistic growth (rate r_T_, carrying capacity c_T_) modified by a smooth extinction term (T-1)/(T+1), which ensures negative growth when T<1. CAR T-cell cytotoxicity is modeled as a saturating function of effector density with killing rate r_K_ and half-saturation constant c_K_. Initial conditions are C_i_(0)=C_0_, C_E_(0)=C_P_(0)=0 and T(0)=T_0_.

### 2.6 Model parametrization and parameter estimation

The ODE model (Equations 2–5) comprises 14 mechanistic parameters (m_I_, m_E_, m_P_, r_A_, r_E_, r_F_, r_R_, a_d_, r_T_, c_T_, r_K_, c_K_, C_0_, T_0_). Based on sensitivity analysis performed in our previous work (21), three parameters with minimal impact on kinetic shape were fixed across all simulations (**Supplementary Material Table S1**): carrying capacity (c_T_=10^12^ cells), memory recall rate (r_R_=0.1 day^-1^), and killing half-saturation (c_K_=10^7^ cells). The memory recall rate lies within the literature range compiled there and is a low-sensitivity parameter that can be varied over several orders of magnitude without noticeable changes in the simulated kinetics. Additionally, in the initial model setup, the three tumor-related parameters were held constant to evaluate the model under uniform assumptions: antigen density (a_d_=10^–6^ cell^-1^), tumor growth rate (r_T_=0.2 day^-1^) and initial tumor load (T_0_ = 10^8^ cells).

To estimate the remaining eight CAR-T specific parameters, we adopted an established multi-step approach, which exploits the mechanistic link between model parameters and the multiphasic kinetics (21). There, we demonstrated that each of the eight segmentation parameters (slopes s_D_, s_E_, s_C_, s_P_ and levels C_min_, C_max_, C_con_, C_per_) is predominantly governed by a specific mechanistic counterpart, establishing an approximately one-to-one correspondence (**Supplementary Figure S2**): in the distribution phase, the initial dose C_0_ determines the initial level, the death rate (m_I_) sets the slope s_D_, and the activation rate r_A_ determines the duration and minimum level (C_min_); in the expansion phase, the proliferation rate (r_E_) controls the expansion slope (s_E_) and the peak magnitude (C_max_), while the killing rate (r_K_) sets the peak (C_max_); during the contraction phase, the decay of effector cells (m_E_) dictates the contraction slope (s_C_) and the memory formation rate (r_F_) determines the end of contraction phase (C_con_); during the persistence phase, the persister death rate (m_P_) governs the long-term slope (s_P_). This correspondence is a first-order approximation rather than a strict bijection: the peak magnitude C_max_ is jointly determined by the proliferation rate r_E_ and the killing rate r_K_, and it is precisely for this reason that r_E_, r_F_ and r_K_ are subsequently refined by numerical optimization rather than obtained analytically.

Using the corresponding analytical approximations (21) we directly translate five segmentation parameters into five mechanistic model parameters (namely, m_I_, m_E_, m_P_, r_A_, C_0_), while the remaining three mechanistic parameters r_E_, r_F_, and r_K_ are optimized using the *NonlinearModelFit* function in *Wolfram Mathematica* with the other segmentation parameters guiding boundaries of the optimization. **Supplementary Material Table S1** provides a comprehensive overview of this estimation methodology, parameter definitions, baseline values, and bounds. Only in a subsequent approach (**Figures 4**, **5**), the three additional tumor parameters (a_d_, r_T_, T_0_) were allowed to vary to capture patient-specific heterogeneity.

## 3 Results

### 3.1 Multiphasic kinetics characterize the patient cohort

We analyzed data from all patients for which sufficient data was available (**Figure 1A**, for clinical data of included and excluded patients see **Supplementary Data Table 1**). The cohort of 46 patients was stratified based on their therapeutic outcome and the costimulatory domain of the CAR construct. In terms of therapeutic outcome, 16 patients finally exhibited disease progression (PD), while 30 patients did not progress (non-PD) during follow-up (**Figure 1B**). The high overall success rate (65%) may result from the selection process and the exclusion of patients with a short follow-up due to unfavorable prognosis. Consistent with this, a descriptive comparison with the 46 excluded patients (**Supplementary Data Table 1**) shows a slightly higher proportion of progression among excluded patients within each costimulatory-domain stratum (CD28: 43.3% vs 34.4%; 4-1BB: 31.2% vs 21.4%) and a shorter median follow-up (CD28: 266 vs 377 days; 4-1BB: 627 vs 772 days); these differences are descriptive, as the formal tests in **Supplementary Data Table 1** contrast CD28 with 4-1BB. Because patients who progress early contribute fewer MFC measurements, the analyzed cohort is expected to be enriched for favorable courses, which may accentuate the associations reported below.

Regarding disease type, 38 patients had aggressive B-cell non-Hodgkin lymphoma (aNHL), 4 mantle cell lymphoma (MCL), 2 indolent B-cell non-Hodgkin lymphoma (iNHL), and 2 B-cell acute lymphoblastic leukemia (B-ALL). Concerning the CAR T-cell product, 32 patients were treated with a product containing a CD28-based costimulatory domain (28 Axi-Cel, 4 Brexu-Cel), while 14 patients received a CAR T-cell product containing a 4-1BB costimulatory domain (10 Tisa-Cel, 4 Liso-Cel). Differences in PFS according to the costimulatory domain (**Figure 1C**, Cox proportional hazards model: HR 0.47, p=0.248) reflect sampling variability rather than a true efficacy difference.

After administration, CAR T-cell kinetics in PB of individual patients followed a multiphasic trajectory (**Figures 1D, E**), with four distinct phases as described before (18, 19, 21, 24): an initial *distribution phase*, which may be absent in some cases, characterized by a reduction in CAR T-cell counts in the PB; an *expansion phase*, marked by rapid proliferation peaking approximately 1-2 weeks post-infusion; a *contraction phase*, defined by a sharp decline over the subsequent 2-6 weeks; and finally, *a persistence phase*, during which cell counts stabilize at lower levels. Using the procedure detailed in the Methods, we segmented the time courses from all 46 patients into these four phases (**Figure 1F**). The overall median kinetics did not significantly differ in the expansion phase between the PD and non-PD groups, while the non-PD group presented a slower contraction phase (**Figure 1G**). Regarding CAR T-cell product, patients treated with a CD28-based costimulatory domain exhibited higher expansion factors from C_min_ to C_max_ but lower long-term persistence compared to 4-1BB-based domain (**Figure 1H**).

### 3.2 Association between CAR T-cell kinetics, toxicity, outcomes and tumor burden

The segmentation of CAR T-cell kinetics allows for the transformation of longitudinal data into eight segmentation parameters (**Figures 1D, E**): the four key levels for each phase (C_min_, C_max_, C_con_, C_per_) and the four observed slopes (s_D_, s_E_, s_C,_ s_P_). We evaluated the associations between therapeutic outcomes and each of these eight segmentation parameters, in addition to CAR T-cell counts measured at various time points during the persistence phase (**Supplementary Figure S3**). Hereby, we focused on patients treated with CAR T-cell products containing a CD28-based costimulatory domain, as they represented the largest cohort and as their expansion kinetics differ from CAR T-cells with a 4-1BB-based costimulatory domain.

The analysis of the expansion and contraction phases revealed a moderate association with patient outcomes (**Figures 2A–D**). We identified a correlation between both a higher peak concentration of CAR T-cells and a steeper slope during the expansion phase and better therapeutic effectivity (**Figures 2A, B**, Cox proportional hazards model: HR 3.83, p=0.021). We applied thresholds that maximized the difference in response between low versus high peak concentrations, and slow versus fast expansion slopes. This separation revealed that a high proportion of patients (83%) with both low peak concentrations and slow slopes later developed PD, against 31% of patients developing PD within the group with high CAR T-cell peak and/or fast expansion. This suggests that stronger CAR T-cell expansion is associated with a better prognosis in patients treated with an anti-CD19 CAR T-cell product with a CD28-based costimulatory domain, thereby confirming previous findings (28). These thresholds, as well as the corresponding contraction-phase cutoffs below, were selected *post hoc* to maximize group separation within this cohort. Given the relatively small subgroup (32 CD28 patients), these threshold-based associations should be regarded as exploratory rather than as predefined cut-offs transferable to other cohorts.

Focusing on the contraction phase, we found that most patients (83%) with both a slower contraction phase and a shallow contraction level C_con_ had non-PD, in contrast to those with a steeper contraction slope and/or a deeper contraction level, of whom 45% had non-PD, (**Figures 2C, D**, Cox proportional hazards model: HR 5.36, p=0.030). We hypothesize that a weaker contraction phase is associated with a higher abundance and persistence of potent effector CAR T-cells.

Investigating the association between CAR T-cell kinetics and toxicity, a trend toward more severe ICAHT (maximum grades of anemia, thrombopenia and neutropenia up to month 1) was observed in patients with faster expansion slopes (**Figure 2E**; U-test, p=0.052 for anemia, p=0.019 for thrombopenia, p=0.078 for neutropenia). No association was found between CAR T-cell peak and cytopenias, nor between CAR T-cell expansion slope or peak with CRS or ICANS (**Supplementary Figure S4A**). We also explored associations between treatment effectiveness and ICAHT (graded by CTCAE), CRS and ICANS severity (graded by ASTCT criteria). Although the PD and non-PD groups showed no difference in cytopenia prior to lymphodepleting chemotherapy (LD), the PD group showed more durable severe ICAHT, with higher grades persisting through months 1 and 3 (**Figure 2F**, **Supplementary Figure S4B**). This suggests that rapid resolution of cytopenia after CAR T-cell administration may be associated with better prognosis. Concerning the occurrence of CRS and ICANS within the first two weeks after infusion, we found no sensible differences between PD and non-PD groups (**Supplementary Figure S4C**).

The association between initial tumor load and response has also been investigated in the past (29). Since data on tumor volume before CAR T-cell therapy was unavailable, we used surrogate markers of tumor proliferation and burden. We found that patients with a worse prognosis tended to have higher LDH levels, higher Ann-Arbor stage, or a higher IPI score before LD (**Supplementary Figure S5AC**).

In summary, our results suggest that strong CAR T-cell expansion, slow CAR T-cell contraction, and shortened ICAHT are associated with favorable therapeutic responses and limited disease status before CAR T-cell therapy.

### 3.3 Mechanistic model with uniform tumor assumptions limits prediction accuracy

Beyond the multiphasic segmentation we developed a mechanistic model of CAR T-cell interactions, formulated in terms of ODEs to achieve a functional understanding of the resulting kinetics. We apply this model to describe the CAR T-cell response in 46 patients included in this study (**Figure 3A**). In brief, the model comprises three CAR T-cell compartments: injected, expanding, and persistent, alongside a tumor cell population. We hypothesize that antigen exposure on tumor cells drives transitions between CAR T-cell compartments. Injected CAR T-cells are activated upon antigen contact and transition to the expanding compartment, where they proliferate and exert cytotoxic effects on tumor cells. These cells further transition to persistent, long-lived and non-proliferative CAR T-cells, which can revert to expansion in an antigen-dependent manner. Tumor cells follow logistic growth and are eradicated by CAR T-cell-mediated cytotoxicity.

Technically, the model is defined by 4 ODEs with 14 mechanistic parameters. To optimally adapt the mechanistic model to the available time course data for each patient, we adopt a multistep parameter estimation strategy developed previously (21). This strategy is based on an approximate analytical correspondence between the eight segmentation parameters of the multiphasic kinetics and the eight mechanistic parameters (**Figure 3A**, **Supplementary Figure S2**): initial CAR T-cell dose, decay rates for the three CAR T-cell types, activation rate of injected CAR T-cells, proliferation rate of expanding CAR T-cells, memory formation rate of persistent CAR T-cells, and cytotoxicity of CAR T-cells against tumor cells. Employing this analytical correspondence, optimal model fits are obtained by minimizing the error between model and data (details in Methods, **Supplementary Figure S2**, **Supplementary Material Table S1**). In this first estimation setup, three tumor-related model parameters (namely initial tumor load (T_0_), tumor growth rate (r_T_), and average antigen density (a_d_); marked in brown in **Figure 3A**) are initially fixed across patients, assuming a uniform tumor profile. The remaining three parameters (marked in gray in **Figure 3A**), namely the tumor carrying capacity (theoretical maximum tumor burden), the activation rate of persistent CAR T-cells, and the average CAR T-cell receptor density, are also assumed to be uniform across patients, but have less impact on the model dynamics.

Applying this strategy, we obtain patient specific model parameters that reproduce individual CAR T-cell kinetics (**Figure 3B**), including a spectrum of responses ranging from tumor relapse to durable remission (**Figures 3C, D**). Using a threshold of 10^5^ simulated tumor cells to define PD, the model closely matches the clinical data on PFS at the population level. This threshold is an operational proxy for a clinically detectable burden and is defined relative to the initial tumor load, which is itself varied in the following sections, so that our conclusions do not depend on its precise value. However, when comparing these predictions with the reported outcomes, a notable discrepancy emerged between the model’s predictions and the observed efficacy (**Figure 3C**). Indeed, although the model correctly predicts the outcome of 29 out of 46 patients, we identify 9 false-negative and 8 false-positive predictions. This suggests that while the model can capture individual trends for the CAR T-cells kinetics, a modeling approach that employs uniform tumor cell parameters across the whole patient population fails to adequately predict the individual tumor responses.

In conclusion, our mechanistic model explains the multiphasic CAR T-cell dynamics with patient-specific CAR T-cell properties, but fails to capture individual clinical outcomes under the assumption of a uniform tumor.

### 3.4 Tumor heterogeneity explains identical CAR T-cell kinetics with variable tumor outcome

Next, we investigated to which extent the heterogeneity of tumor specific features can better explain clinical outcomes. Analyzing our model revealed that the three tumor specific model parameters (initial tumor load, tumor growth rate, and antigen density) directly correlate with the CAR T-cell expansion peak without affecting other aspects of the CAR T-cell dynamics. Increasing one of these parameters leads to parallel kinetics with higher CAR T-cell peaks, and vice versa (**Figures 4A–C**).

We also revealed that changes in one tumor parameter can be compensated by opposing changes in another, yielding indistinguishable CAR T-cell kinetics. Such inverse parameter dependencies allowed us to fit individual time courses equally well, despite reflecting different underlying tumor characteristics (**Figures 4D-F**; top). We reason that identical CAR T-cell kinetics can be explained by different tumor characteristics: increased tumor load with decreased tumor growth rate, increased tumor load with decreased antigen density, and increased tumor growth rate with decreased antigen density (and vice versa). This also indicates that within the model CAR T-cell expansion results from the total antigen exposure, measured as the convolution of tumor mass, growth rate and antigen density.

Simulated tumor dynamics resulting from these different parameterizations showed that incorporating tumor heterogeneity can produce identical CAR T-cell kinetics while explaining variable tumor outcomes (**Figures 4D-F**, bottom). Specifically, higher tumor load (**Figures 4D, G**) or antigen density (**Figures 4E, H**), each linked with decreased tumor growth rate, correlated with fewer progressions and improved therapeutic responses. The combination of increased antigen density and decreased tumor load also led to slightly improved tumor outcome (**Figures 4F, I**). These findings suggest that tumor mass, growth rate, and antigen density all contribute to CAR T-cell antigen-mediated expansion and transitions, thereby determining therapy response.

In conclusion, the mechanistic model successfully explains how identical CAR T-cell kinetics can lead to divergent tumor outcomes. Therefore, incorporating patient-specific tumor characteristics is essential to account for individual therapeutic outcomes.

### 3.5 Patient-specific tumor characteristics reconcile CAR T-cell kinetics with observed outcomes

Assuming uniform tumor parameters across all patients led to 17 misclassifications (**Figure 5A**). To explicitly include tumor heterogeneity in the model fitting, we systematically varied tumor growth rate and initial tumor load within biologically plausible ranges (**Supplementary Material Table S1**). Specifically, three discrete values for the initial tumor load and four values for the growth rate were considered, resulting in 12 possible (T_0_, r_T_) parameter pairs. For each pair, we optimized the antigen density a_d_ independently to achieve the best fit to the individual CAR T-cell kinetics (**Supplementary Material Table S1**), yielding 12 equally good fits per patient that reproduced the CAR T-cell kinetics while generating a range of tumor outcomes.

To refine the predictions, we identified (T_0_, r_T_) parameter pairs that best aligned with clinical outcomes. For non-PD patients, we selected parameter sets yielding a non-PD tumor outcome while minimized the error between model-predicted and observed CAR T-cell kinetics. This approach successfully described all 30 non-PD patients. For PD patients, we chose parameter pairs reproducing relapse and approximating the observed relapse time, correctly classifying 12 out of 16 PD patients. The remaining 4 were consistently misclassified as non-PD.

This refined approach substantially improved prediction accuracy, producing 42 correct classifications and only 4 false negatives (**Figure 5B**). It suggests that incorporating patient-specific estimates of tumor growth rate and initial tumor load prior to CAR T-cell infusion leads to better model calibrations and more accurate long-term response predictions. Notably, varying only one of these parameters at a time while keeping the other fixed resulted in similar misclassification rates as the first uniform approach, with 7 to 8 false predictions (**Supplementary Figures S6A, B**). Additionally, expanding the parameter range did not further improve predictive accuracy (**Supplementary Figure S6C**). Although more complex fitting strategies could be employed, this systematic grid search is sufficient to demonstrate that the solution is non-unique and that specifying the tumor parameters (T_0_, r_T_) effectively constrains the degrees of freedom to retrieve the correct clinical outcome. This confirms that the current limitation arises from the lack of patient-specific tumor data rather than the methodological approach.

Beyond individual patient responses, the 12 (T_0_, r_T_) parameter pairs represent distinct tumor profiles, ranging from small, slow-growing tumors to large, aggressive ones (**Figure 5C**). Despite similar CAR T-cell expansion kinetics (**Figure 5D**), the corresponding tumor responses varied substantially: patients with small, slow-growing tumors had the best outcomes, while those with large, fast-growing tumors had the poorest prognosis (**Figure 5E**). At the population level, these different tumor characteristics had a strong impact on PFS, with small, slow-growing tumors leading to a predicted PFS of 90% at 18 months, while large, fast-growing tumors were associated with a significantly lower PFS of 50% at the same time point (**Figure 5F**).

Given that tumor growth rate and initial tumor load are critical for predicting patient outcomes, we investigated whether clinically available patient-specific tumor characteristics could serve as surrogate markers for these parameters. Hypothesizing that Ann-Arbor stage or IPI score reflect total tumor burden, we analyzed their correlation with the best-fit parameter for initial tumor load and tumor growth rate (**Supplementary Figures S6D, E, G, H**) but found no significant correlation. Additionally, since LDH levels before LD are considered a marker of cell turnover, we assessed their correlation as well (**Supplementary Figures S6F, I**), yet no significant correlation was observed.

In summary, patient-specific tumor characteristics improve long-term outcome prediction in CAR T-cell therapy; however, surrogate markers poorly capture these parameters. This underscores the need to integrate individualized tumor profiling into predictive models to enhance therapeutic decision-making.

## 4 Discussion

Our study presents an integrative framework to characterize patient-specific outcome in CAR T-cell therapy by combining multiphasic kinetic segmentation with mechanistic mathematical modeling. We show that early kinetic features, such as expansion peak and slope, as well as contraction dynamics, correlate with relapse risk and offer useful prognostic markers. However, kinetic patterns alone are insufficient for individualized predictions. By fitting a CAR T-cell – tumor interaction model to longitudinal data, we demonstrate that similar CAR T-cell kinetics can result from markedly different tumor profiles, emphasizing the importance of tumor-specific parameters. Importantly, our findings underscore that current surrogate marker like LDH levels or Ann-Arbor stage cannot improve individualized predictions. This work advances the field by providing a quantitative framework that, when informed by detailed tumor characteristics, supports therapeutic decision-making in CAR T-cell therapy.

Early identification of treatment failure or severe toxicity remains a major clinical need, given the poor prognosis of patients with early relapses and the limited effectiveness of salvage therapies. Although all eligible patients currently proceed to CAR T-cell infusion, early prediction of relapse within the first three months could create a critical window for intervention. Ongoing efforts, including PET-based imaging (30) and cfDNA-based residual disease monitoring (31), aim to address this need and may be complemented by early CAR T-cell kinetic features observed predominantly during the first month of therapy. However, unlike chemically defined drugs, CAR T-cells are living therapeutics with substantial inter- and intra-product heterogeneity, shaped by the tumor and immune microenvironment. Despite this complexity, by flow cytometry we observed a consistent four-phase kinetic pattern across patients, with product-specific differences linked to the costimulatory domain. Our findings are consistent with prior studies showing that early expansion (C_max_, AUC_0_-_28_) predicts response across various products, including axi-cel, brexu-cel, liso-cel, and tisa-cel (28, 32–36). Our analysis extends beyond established correlations by identifying the contraction phase as a critical determinant of response; a ‘slow contraction’ phenotype was strongly associated with durable remission, suggesting that the rate of effector cell decline is as prognostically relevant as the expansion peak.

We also identified an association between faster expansion and increased hematotoxicity. Moreover, in our cohort, prolonged ICAHT was linked to inferior PFS, consistent with prior reports (37). While the causality between CAR T-cell expansion and hematotoxicity remains uncertain, their co-occurrence may restrict subsequent treatment options. In contrast, we found no significant correlation between kinetic parameters and CRS incidence, although there was a trend toward increased CRS severity with faster expansion. This may reflect the limitations of clinical grading in capturing the full extent of systemic inflammation (38). Additionally, higher CAR T-cell expansion has been associated with more frequent use of immunosuppressive agents such as tocilizumab and glucocorticoids (39).

To move beyond descriptive kinetic analyses, we applied a mechanistic model based on CAR-T-tumor cell interactions. While more complex formulations for tumor-immune interactions exist, such as the ‘Fractional Kill’ hypothesis (40, 41), we opted for a saturation term (Holling Type II) to maintain structural identifiability given the absence of longitudinal tumor data. While the model reproduced individual CAR T-cell kinetics and PFS risk at the population level, it lacked predictive power at the individual level when tumor parameters were held constant. The model revealed that varying combinations of tumor burden, proliferation rate, and antigen density can produce indistinguishable CAR T-cell kinetics, highlighting the need to incorporate patient-specific tumor characteristics. However, neither Ann-Arbor stage, IPI score nor LDH, quantified at a single pre-infusion time point, showed correlation with the estimated tumor growth rate, initial tumor load, or antigen density, despite their established prognostic relevance (42). Therefore, the estimated tumor parameters remain to be validated, which is a prerequisite for individual outcome predictions. Serial quantification of tumor burden measured in parallel with CAR T-cell kinetics would provide the information needed to constrain the tumor parameters uniquely.

This challenges the utility of these markers for individual prognosis and highlights the need for more precise surrogates. Alternative serum biomarkers—such as thymidine kinase, β2-microglobulin, alkaline phosphatase, IL-6, and IL-10—may better reflect tumor proliferative activity (43–46). Similarly, metabolic tumor volume or maximum tumor diameter may outperform Ann-Arbor stage in quantifying tumor burden (47). The clinical assessment of antigen density remains particularly challenging, despite its mechanistic relevance. Our framework may be especially suited to diseases such as multiple myeloma, where serum monoclonal protein levels provide a clinically accessible and reliable surrogate for tumor burden (48), offering a potential avenue for real-time model calibration and personalized prediction.

Regarding model validation, we acknowledge that our approach differs from standard predictive/pharmacokinetic modeling. Instead of training a statistical classifier on a hold-out set, we solved the mechanistic ‘inverse problem’ to map observed kinetics to hidden tumor states. The reported high classification accuracy therefore reflects mechanistic concordance. In other words, it underpins the ability of the structure to reconcile blood kinetics with clinical outcomes when informed by plausible tumor parameters, rather than a validated prospective prediction. This retrospective analysis validates the biological framework and highlights the necessity of integrating future tumor quantification (e.g., ctDNA) in lymphoma treatment to fully unlock prospective applications.

In conclusion, while our model establishes a foundation for individualized outcome prediction in CAR T-cell therapy, its clinical translation will require integration of accessible, tumor-specific biomarkers. Further refinement of these models may enhance their prognostic and therapeutic utility in real-world settings.

## Data Availability

The original contributions presented in the study are included in the article/**Supplementary Material**. Further inquiries can be directed to the corresponding author.
