# Supplementary material for: Model-based integration of chimeric antigen receptor T-cell kinetics and tumor features to predict therapeutic outcomes in patients with B-cell malignancies
Source: Front Immunol. 2026 Oct 2;17:1927857. doi: 10.3389/fimmu.2026.1927857 (PMC13672575; doi:10.3389/fimmu.2026.1927857)
Supplement: Supplementary file 1 [file DataSheet1.pdf]

Supplementary information for "Model-based integration of chimeric antigen receptor T-cell kinetics and tumor features to predict therapeutic outcomes in patients with B-cell malignancies"

Jonas Schadt[1*], Maximilian A Röhnert[1*], Paul Matthiensen[1], Uta Oelschlägel[1], Martin Bornhäuser[1], Frank Kroschinsky[1], Raphael Teipel[1], Ingmar Glauche[2], Malte von Bonin[1], Artur César Fassoni[2,3]

Table S1. Description of model parameters, estimation strategy, baseline values, and bounds.

Parameter	Meaning	Estimation Strategy	Units	Baseline	Bounds/Condition
Fixed parameters (low sensitivity parameter, uniform for all patients)					
r_R	Memory recall rate	Fixed	day^{-1}	0.1	Fixed
c_T	Tumor carrying capacity	Fixed	cells	10^{12}	Fixed
c_K	Killing half-saturation constant	Fixed	cells	10^7	Fixed
CAR T-cells parameters (fitted from multiphasic segmentation, see Fig. S1)					
C_0	Initial CAR-T cell count in PB	Fitted value derived from CAR T-cell dose	cells	Patient-specific	C_0 = CAR T-cell dose
m_I	Decay rate of injected CAR-T cells	Fitted value derived from slope s_D	day^{-1}	Patient-specific	$m_I = -s_D$
m_E	Decay rate of effector CAR-T cells	Fitted value derived from slope s_C	day^{-1}	Patient-specific	$m_E = -s_C$
m_P	Decay rate of persister CAR-T cells	Fitted value derived from slope s_P	day^{-1}	Patient-specific	$m_P = -s_P$
r_A	Activation rate of injected CAR-T cells	Fitted value derived from (t_{min},C_{min}) and slopes s_E, s_D	day^{-1}	Patient-specific	solution of $(s_E-s_D)=r_A*\exp(t_{min}(r_A+s_E-s_D))$
r_E	Proliferation rate of effector CAR-T cells	Optimized (bounds derived from slopes s_E, s_C)	day^{-1}	Patient-specific	$r_E \in [0.5(s_E-s_C), 2(s_E-s_C)]$
r_F	Memory formation rate	Optimized	day^{-1}	Patient-specific	$r_F \in [10^{-6}, 0.5]$
r_K	Max. cytotoxicity rate of CAR-T cells	Optimized	day^{-1}	Patient-specific	$r_K \in [0.1, 2.5]$
Tumor cells parameters (Fixed in Fig 3 / Varied in Figs 4-5)					
a_d	Tumor antigen density	Fixed in Fig 3 / Varied in Figs 4-5	$cell^{-1}$	10^{-6}	Fixed / Grid Search $\log_{10}(a_d)\in[-10,-1]$
r_T	Tumor growth rate	Fixed in Fig 3 / Varied in Figs 4-5	day^{-1}	0.2	Fixed / Grid Search $r_T\in\{0.2,0.3,0.4,0.5\}$
T_0	Initial tumor burden	Fixed in Fig 3 / Varied in Figs 4-5	cells	10^8	Fixed / Grid Search $T_0\in\{10^7,10^{7.5},10^8\}$

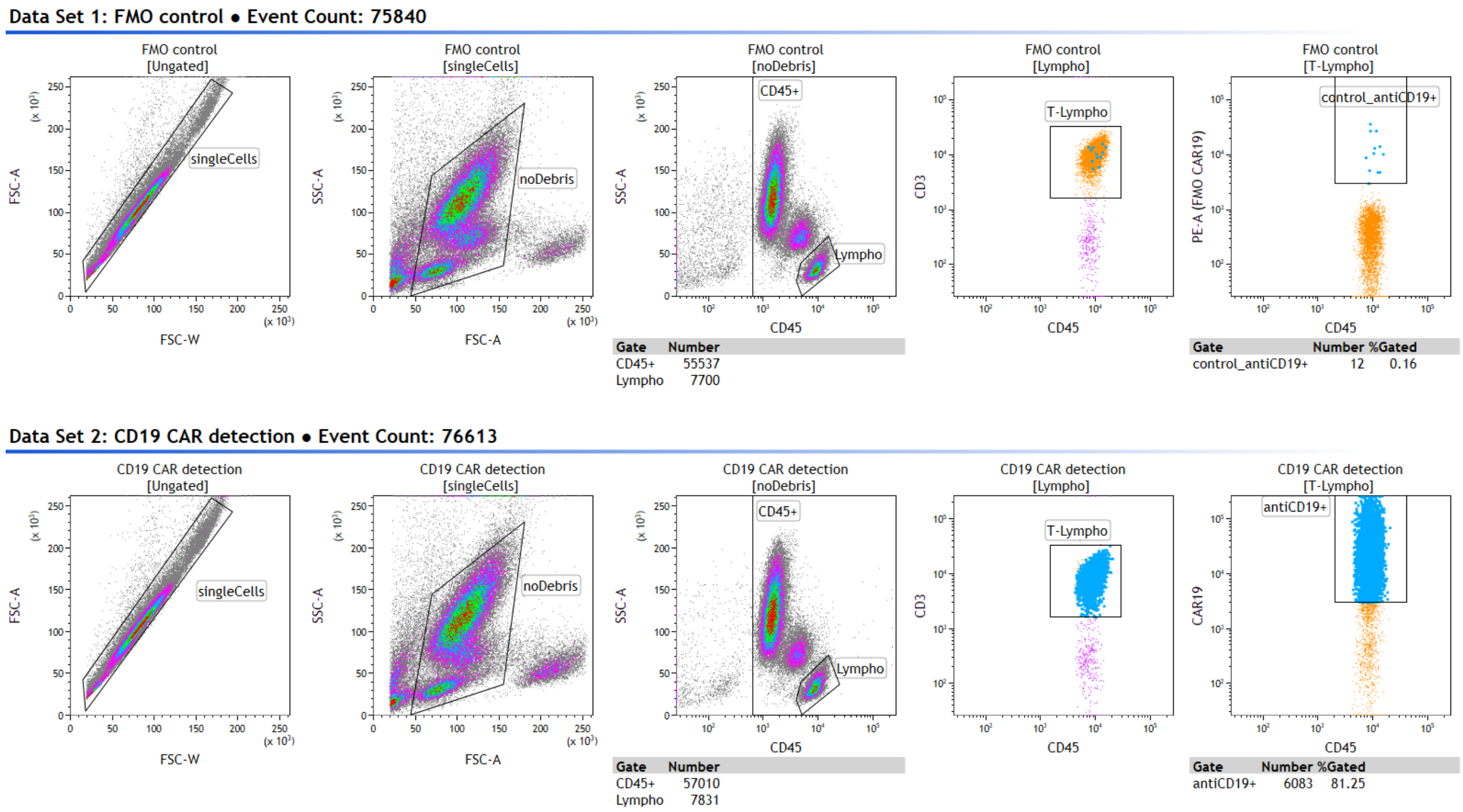

Figure S1. MFC gating strategy. After exclusion of debris and doublets, CD45^{+} cells (leukocytes) and lymphocytes are identified in the SSC/CD45 plot. Within the lymphocyte population, T-lymphocytes are defined based on CD3 positivity, from which CAR T-cells are subsequently identified. According to the fluorescence minus one (FMO) concept, the sample in Data Set 1 does not contain the biotinylated CD19-based CAR detection reagent and therefore serves as the control measurement. Gates in all plots are defined based on this FMO sample and then applied identically to Data Set 2 (which includes the biotinylated CD19-based CAR detection reagent). In some cases, CAR T-cells are already detected in the FMO control (false positives). This fraction is subtracted from the CAR T-cell proportion in Data Set 2 to obtain the true CAR T-cell frequency in the sample.

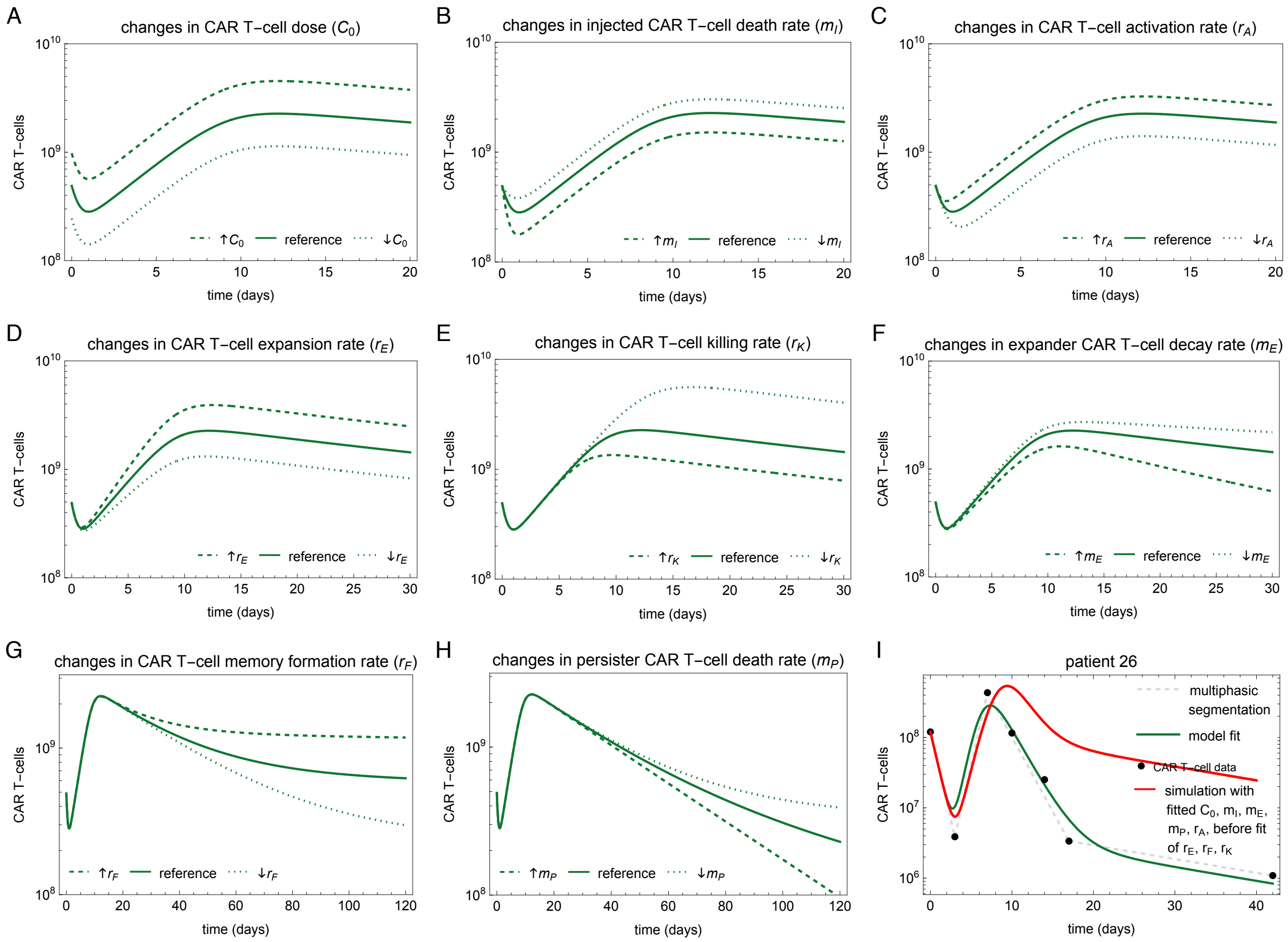

Figure S2. Mechanistic parameters shape de multiphasic CAR T-cell kinetics enabling multistep parameter estimation strategy. (A-H) Each CAR-T cell mechanistic parameter has distinct effects on the shape of the simulated multiphasic kinetics. (A) Changes in the simulated CAR T-cell dose C_0 only translates the simulated multiphasic kinetics up and down. (B) The death rate m_I of injected CAR T-cells only changes the distribution slope. (C) The activation rate r_A only changes the duration of distribution phase. (D) The expansion rate r_E only changes the expansion slope and the peak. (E) The killing rate r_K only changes the peak. (F) The decay rate m_E only changes the contraction slope. (G) The memory formation rate r_F only changes the end of contraction phase. (H) The death rate m_P only changes the persistence slope. (I) This one-to-one correspondence between eight mechanistic parameters and eight shape features of the multiphasic kinetics enables a multistep parameter estimation strategy. First, the CAR T-cell data (dots) is segmented (dashed gray lines) with an automated routine (Methods) providing eight patient-specific values corresponding to the shape features (slopes s_D, s_E, s_C, s_P and levels C_{min}, C_{max}, C_{con}, C_{per}). Then, five mechanistic parameters (C_0, m_I, m_E, m_P, r_A) are directly calculated from these values using analytical approximations derived previously[1] (Table S2). This first step allows to simulate multiphasic kinetics (red) with same slopes as the multiphasic segmentation, but different peaks and contraction levels, but using constant and uniform values for the other three CAR T-cell mechanistic parameters (r_E, r_F, r_K). These three parameters are then fitted using the *NonlinearModelFit* function in *Wolfram Mathematica* (bounds in Table S2) producing the model fit that minimizes residuals (green). During this estimation procedure, the other six model parameters are held constant and uniform for all patients (Table S2); the tumor parameters (r_T, a_d, T_0) are varied in a subsequent approach (Figs. 4,5).

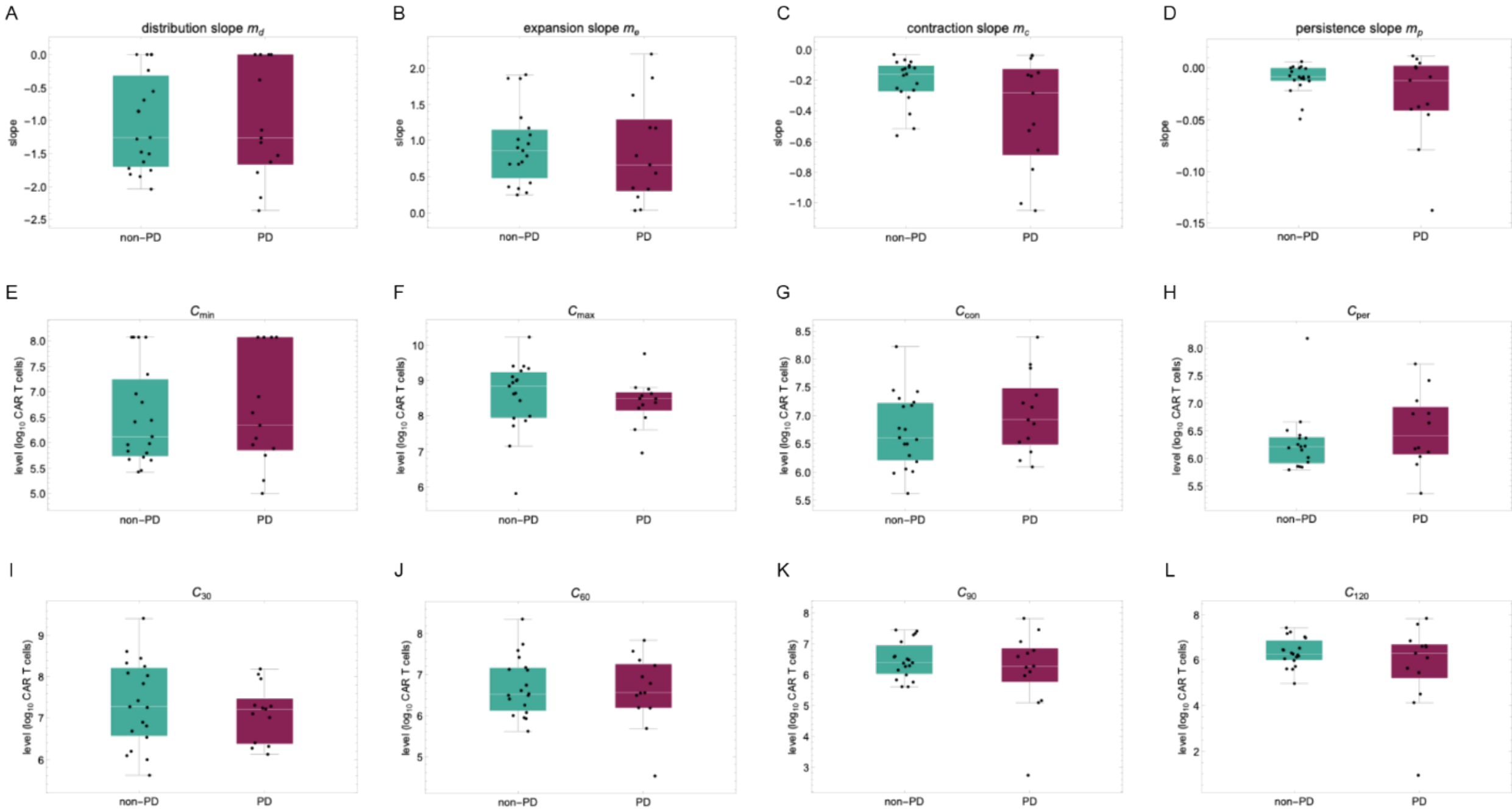

Figure S3. Associations between CAR T-cell kinetics and effectivity. Comparison of kinetic parameters between PD and non-PD groups, including slopes s_D, s_E, s_C, s_P (A-D), key levels C_{min}, C_{max}, C_{con}, C_{per} for each phase (E-H) and CAR T-cell counts C_{30}, C_{60}, C_{90}, C_{120} measured at days 30, 60, 90, 120 after administration (I-L).

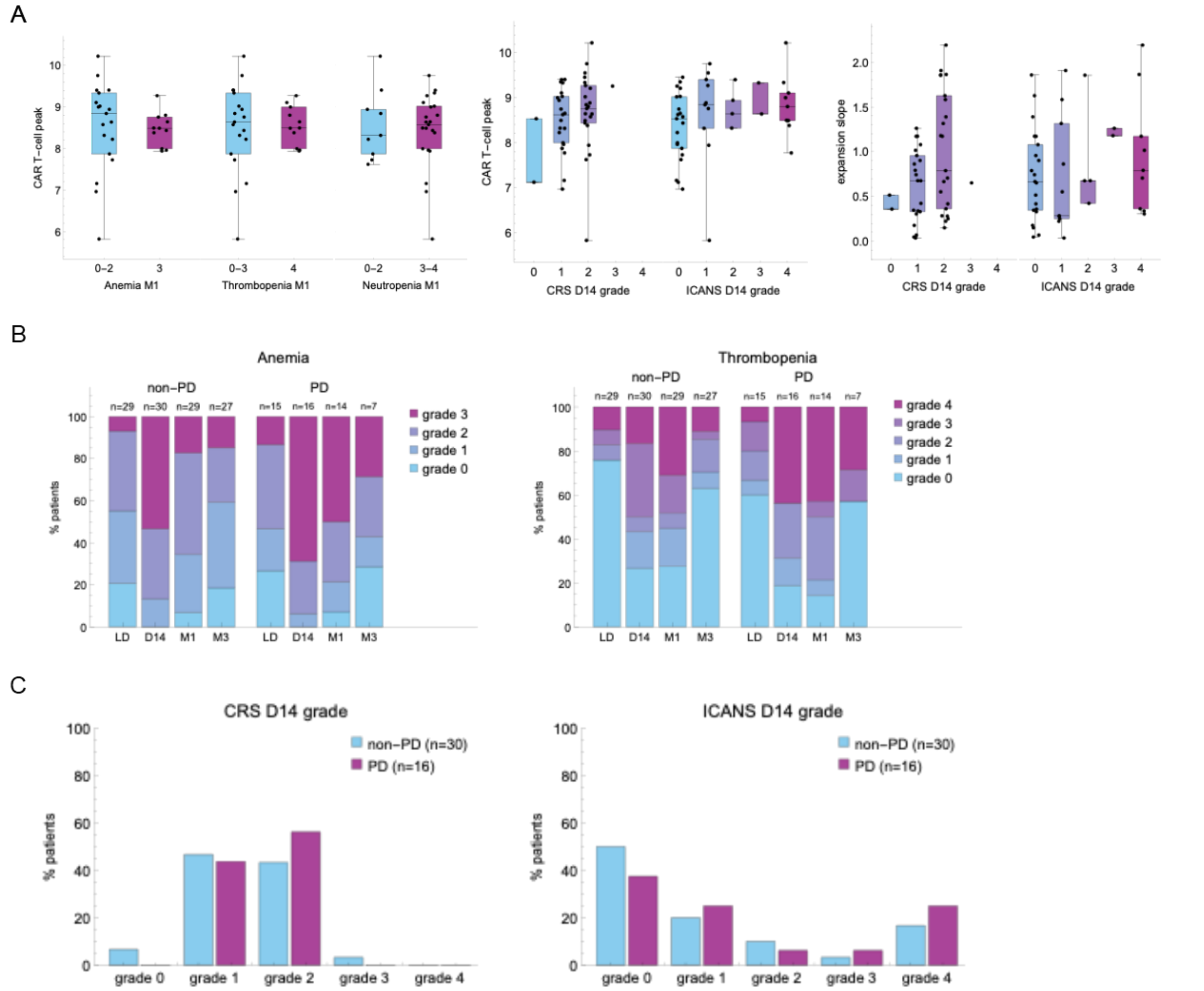

Figure S4. Associations between CAR T-cell kinetics, toxicity, and effectivity. (A) Association between CAR T-cell peak and ICAHT (left), CAR T-cell peak and CRS/ICANS (middle), and CAR T-cell expansion slope and CRS/ICANS (right). B) Evolution of maximum grades anemia and thrombopenia over time in non-PD and PD groups, with the PD group exhibiting persistence of higher severity up to month 3. (C) Incidence and severity of CRS and ICANS show no sensible differences between PD and non-PD groups.

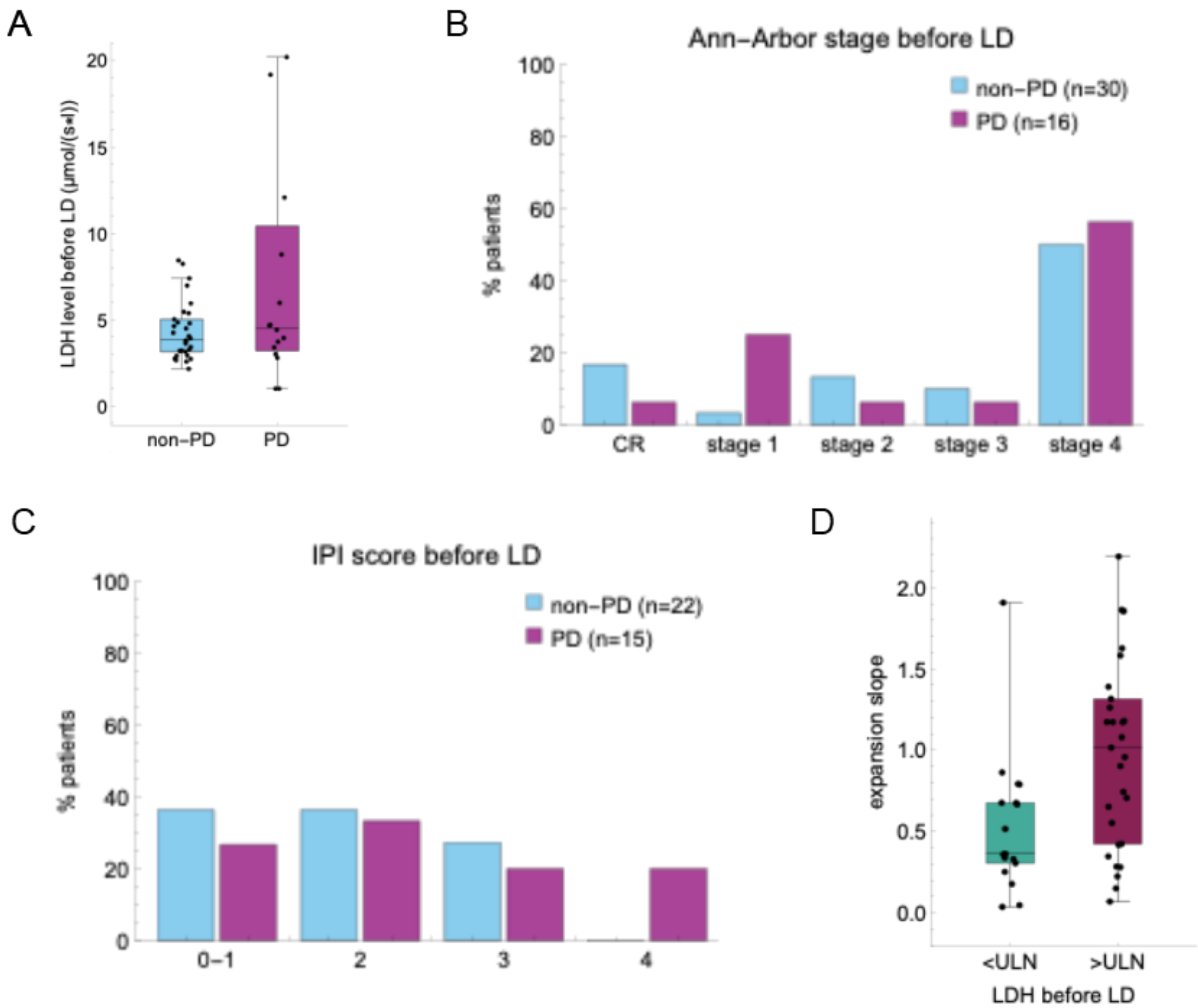

Figure S5. Associations between tumor surrogates, effectivity and CAR T-cell kinetics. (A, B, C) surrogate markers of tumor proliferation and burden (LDH, Ann-Arbor stage, and IPI score before LD) are higher in the PD group, indicating a potential association with worse prognosis. (D) Patients with higher LDH levels showed a tendency of increased CAR T-cell expansion slope, suggesting that more aggressive tumors fuel CAR T-cell expansion (U-test, p=0.011).

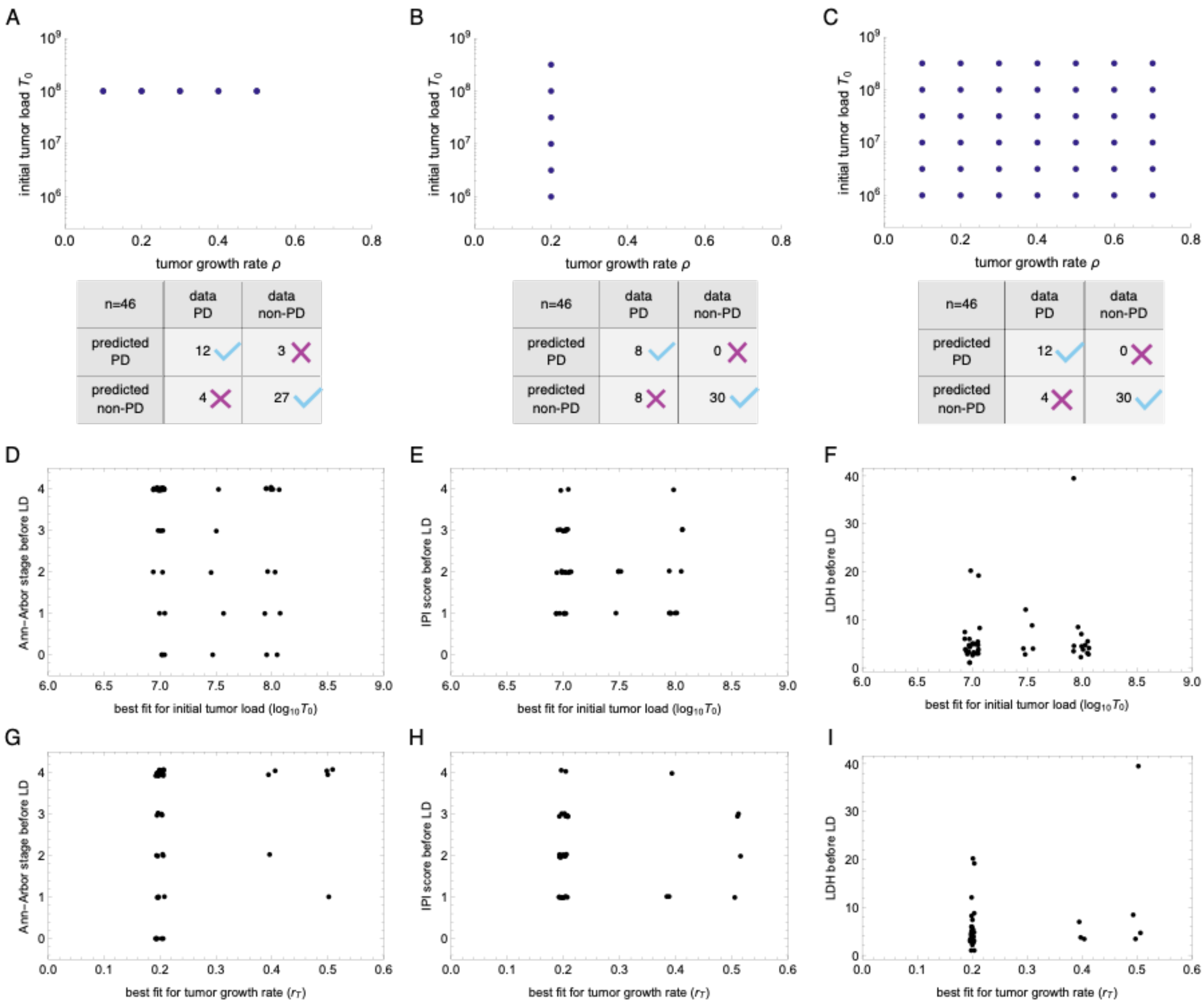

Figure S6. Evaluating model performance and correlation with patient-specific tumor characteristics. (A-C) Impact of different assumptions for varying the parameter pair (T_0, rT) on model predictions, with corresponding confusion matrices. Incorporating heterogeneity in either tumor growth rate (A) or initial tumor load (B) alone does not improve model accuracy compared to using uniform parameter values (Fig. 5A). Allowing both parameters to vary across a broad biologically plausible range improves predictions (C), but a moderate and more realistic range (Fig. 5B) achieves the same accuracy. (D-I) Correlation plots between patient-specific tumor markers and model-derived parameters. To better visualize individual data points, slight noise was added to the x- and y-axes for cases where multiple patients shared the same parameter values. No significant correlation is observed between the best-fit initial tumor load and Ann-Arbor stage (D), IPI score (E), or LDH levels (F) before LD. Similarly, no correlation was observed between the best-fit tumor growth rate and Ann-Arbor stage (G), IPI score (H), or LDH levels (I) before LD.

References

1. Santurio DS, Barros LRC, Glauche I, Fassoni AC. Mathematical modeling unveils the timeline of CAR-T cell therapy and macrophage-mediated cytokine release syndrome. PLoS Comput Biol 2025;21(4):e1012908.
